# A validity study of the rapid emergency Triage and treatment system for children

**DOI:** 10.1186/s13049-021-00832-2

**Published:** 2021-01-23

**Authors:** Siv Steinsmo Ødegård, Thuy Tran, Lars E. Næss-Pleym, Kari Risnes, Henrik Døllner

**Affiliations:** 1 Department of Pediatrics, St. Olavs University Hospital, Trondheim, Norway; 2 Department of Circulation and Medical Imaging (ISB), Norwegian University of Science and Technology (NTNU), Trondheim, Norway; 3 Department of Clinical and Molecular Medicine (IKOM), Norwegian University of Science and Technology (NTNU), Trondheim, Norway; 4 Department of Emergency Medicine and Pre-hospital Services, St. Olavs University Hospital, Trondheim, Norway

**Keywords:** Triage, RETTS-p, Validity, Pediatric emergency care

## Abstract

**Background:**

The Scandinavian Rapid Emergency Triage and Treatment System-pediatric (RETTS-p) is a reliable triage system that includes both assessment of vital parameters and a systematic approach to history and symptoms. In Scandinavia, the system is used in most pediatric emergency departments (PED). We aimed to study the validity of RETTS-p.

**Methods:**

We conducted a study based on triage priority ratings from all children assessed in 2013 and 2014 to the PED at St. Olavs University Hospital Trondheim, Norway. Patients were assigned one of four priority ratings, based on the RETTS-p systematic evaluation of individual disease manifestations and vital parameter measurements. In the absence of a gold-standard for true disease severity, we assessed whether priority ratings were associated with 3 proxy variables: 1) hospitalization to the wards (yes vs. no), 2) length of hospital stay (≤ mean vs. > mean, and 3) referral to pediatric intensive care (yes vs. no). We further compared priority ratings with selected diagnoses and procedure codes at discharge.

**Results:**

Six thousand three hundred sixty-eight children were included in the study. All analyses were performed in the entire population and separately in pediatric sub-disciplines, medicine (*n* = 4741) and surgery (general and neurosurgery) (*n* = 1306). In the entire population and the sub-disciplines, a high priority rate was significantly associated with hospitalization to wards, a longer hospital stay and referral to the pediatric intensive care unit compared to patients with low priority. We observed a dose-response relationship between increased triage code level and indicators of more severe disease (p-trend < 0.001). For the same three proxy variables, the sensitivity was 54, 61 and 83%, respectively, and the specificity 66, 62 and 57%, respectively. Subgroup analyzes within the most common complaints, demonstrated that more severe conditions were higher prioritized than less severe conditions for both medical and surgical patients. Overall, children with surgical diagnoses attained lower priority ratings than children with medical diagnoses.

**Conclusions:**

RETTS-p priority ratings varies among a broad spectrum of pediatric conditions and mirror medical urgency in both medical and surgical disciplines. RETTS-p is a valid triage system for children as used in a university hospital setting.

## Background

In hospital emergency departments (ED), it is important to ensure that the sickest patients are evaluated and treated first. To undertake effective prioritization, several triage systems have been developed [1]. To ensure the best outcome for the patients, and accurate use of resources, it is pivotal to find a triage system which can easily be used, detects ill patients and prioritize the patients correctly [2]. Detecting quickly if patients should be hospitalized will also lead to less waiting for remaining patients [2].

To help prioritizing patients correctly, the  pediatric ED (PED) at St. Olavs University Hospital in Norway implemented the pediatric version of the rapid emergency triage and treatment system (RETTS-p) in 2012 [3]. RETTS was developed at Sahlgrenska University Hospital, Sweden [4]. A pediatric version (RETTS-p) was later developed, which has been increasingly used internationally [4–7]. It is a triage system that together with vital parameter (VP) measurements also is based on clinical information with the most common emergency signs and symptoms (ESS) among children [8].

The most important function of a triage system is to ensure that children in need of medical and surgical interventions are rapidly recognized [9]. It has therefore been argued that it is more crucial that it has a high sensitivity compared to a high specificity [10]. This is especially true for certain patients groups, e.g. children with chronic illness, as studies have shown that they more often are under-triaged compared to otherwise healthy children [11]. As RETTS-p includes independent scores for clinical symptoms related to specific conditions as well as for VPs [8], it might be more sensitive for detecting children in need of hospital admission [1].

Triage tools are usually evaluated by its reliability and validity [1, 5–7, 12]. Others [5, 6] and we [1] have previously found RETTS-p to have a high reliability, which means that it has a high intra- and interrater consistency. The validity of a triage tool refers to the level it can foretell the true urgency of a condition presenting in the ED [12]. The adult version of RETTS have been found to have a high validity [7, 12], but the validity of RETTS-p has not been studied yet. To address this, we used registry data from a large Norwegian PED, and assessed the validity of RETTS-p in a University hospital setting.

## Methods

A population-based study was conducted at the PED at St. Olavs University Hospital, Trondheim, Norway with the aim to validate RETTS-p. This PED is the only in Sør-Trøndelag county and provides emergency care for a population of approximately 58,000 children aged < 16 years old, of whom 18,000 are less than 5 years of age (Statistics Norway, 2014). Most children are referred for evaluation from general practitioners or physicians at the municipal emergency departments, but some children with chronic diseases and children with suspect sexual abuse may come directly to the PED without prior medical evaluation. Children with all types of medical complaints are received, but multi-traumatized and those with severely hampered vital functions are usually received at the main (adult) ED. All children at the PED are registered in a database with contact information, systematic clinical information and triage priority levels. For 2013 and 2014, this registry was linked to data from the patient administration system (PAS) with relevant International classification of disease, 10th revision (ICD-10) diagnoses, procedure codes and other patient administrative data.

### The rapid emergency Triage and treatment system pediatrics (RETTS-p)

Triage with RETTS-p has been described previously [1]. In the present study, we used a Norwegian version of the RETTS-p (version 1.2), which has been translated and adapted to Norwegian conditions. Briefly, the system combines measurements of traditional VPs and evaluation of individual ESS [8]. Forty-two ESS algorithms cover most pediatric complaints [8]. Each ESS algorithm includes recommendations for initial basic evaluation and treatment to be initiated before the PED doctor arrives, such as oxygen treatment. The VPs include open airway, respiratory rate, oxygen saturation, heart rate, alertness level as measured by the Glasgow Coma Scale (GCS), and temperature. The VP priority levels are age adjusted, and heart rate is corrected for fever [1]. At the PED at St. Olavs Hospital the children are triaged according to the RETTS-p at arrival [3]. PED nurses score both VPs and ESSs in one of four priority levels. The final triage priority rating is determined as the highest level from the VP and ESS ratings (red = immediate, orange = within 20 min, yellow = within 2 h, green = within 4 h, blue = no priority, gray = external tasks, brown = social pediatrics and purple is sexual assault unit). Re-triage is done if a change in the child’s clinical condition requires it, and at the latest if the PED doctor has not assessed the patient according to its priority level [3].

### Statistical analysis

In the absence of a gold standard, we assessed the validity of RETTS-p by assessing associations between triage priority levels (red, orange, yellow and green) with three proxy variables for severity: 1) hospitalization to the wards (yes vs. no), 2) a long hospital stay (≤ mean vs. > mean), and 3) referral to the Pediatric Intensive Care Unit (PICU) (yes vs. no). We further compared priority ratings in selected diagnoses and procedure codes at discharge, in order to study if more severe conditions/diagnoses were assigned higher triage ratings than less severe conditions/diagnoses. These analyses were performed for the entire population and separately for medicine and surgery (general and neurosurgery). We calculated sensitivity (proportion of high triage ratings (red + orange) in hospitalized children, children with long hospital stay and children referred to PICU, specificity (proportion of low triage ratings (yellow + green) in children treated as outpatients, children with a short hospital stay and children not referred to PICU), overtriage (proportion of high triage ratings among non-hospitalized children, children with short hospital stay and children not referred to PICU) and undertriage (proportion of low triage ratings in hospitalized children, children with long hospital stay and children referred to PICU). We used Pearson χ^2^-test (univariate analyses) and logistic regression (multivariate analyses) to compare groups. In logistic regression analyses the data was adjusted for age (in years). SPSS Statistics version 23 was used for the analyses.

## Results

**Fig. 1:**
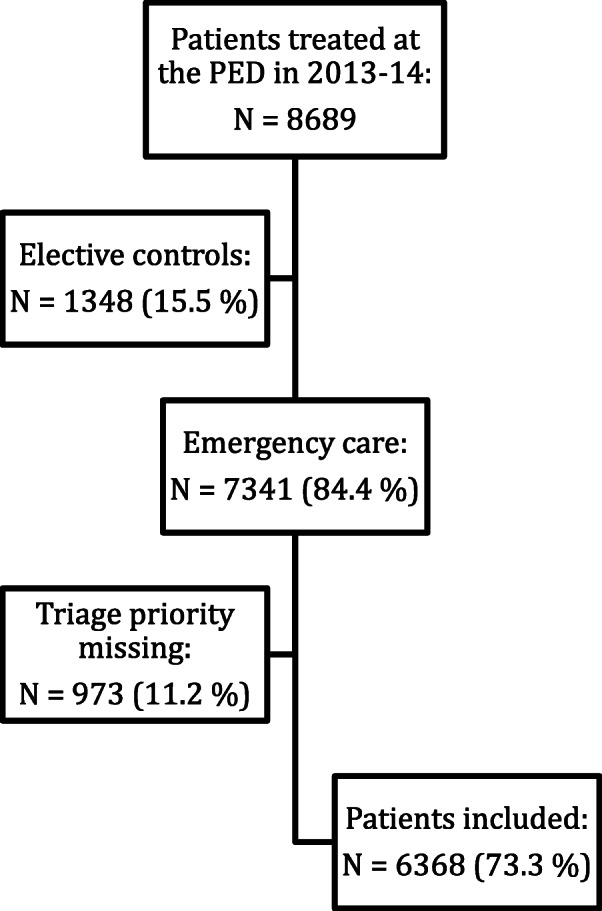
The study population

During the two-year period 2013 and 2014, 8689 children aged 0–16 years old were treated at the PED at St. Olavs Hospital. Elective control patients (*n* = 1348) and children where triage priority level information was missing (*n* = 973) were excluded from the study (Fig. 1). Hence, in all 6368 children were included in the data analyzes, among whom 74% had pediatric medical, 15% surgical, 6% neurosurgical, 5% orthopedic and 0.5% had other complaints. In the entire population 9% of patients had a red triage, 34% had an orange triage, 38% had a yellow triage and 19% had a green triage (Table 1). The median age was 2 years (SD 5 years) (data not tabulated). Pediatric medical patients were more likely to have a high priority compared to the other specialties (Table 1).

**Table 1:** Triage priority levels among the entire population (*n* = 6368) by pediatric disciplines, and selected ESS^a^ codes, ^b^ICD-10 diagnoses and selected procedure codes

	Triage priority ratings				
	Red	Orange	Yellow	Green	Total N
**Entire population (%)**	586 (9)	2179 (34)	2397 (38)	1206 (19)	6368
Pediatric Medicine (%)	538 (11)	1628 (34)	1688 (36)	873 (19)	4727
Child Surgery* (%)	28 (3)	339 (36)	426 (45)	162 (17)	955
Child Neurosurgery* (%)	6 (2)	94 (27)	154 (44)	97 (28)	351
Child Orthopedics* (%)	13 (4)	105 (35)	116 (39)	66 (22)	300
Child Ear-Nose-Throat (%)	1 (3)	12 (39)	12 (39)	6 (19)	31
Child Ophthalmology (%)	0 (0)	1 (25)	1 (25)	2 (50)	4
**ESS-codes**					
Airway problem (104 + 144) (%)	300 (17)	684 (39)	584 (33)	186 (11)	1754
Abdominal pains (106) (%)	29 (3)	311 (31)	483 (48)	184 (18)	1007
Vomiting and diarrhea (110) (%)	15 (3)	172 (34)	177 (35)	146 (29)	510
Head injury (130) (%)	8 (3)	61 (20)	142 (47)	93 (31)	304
Headache (119) (%)	2 (2)	71 (53)	24 (18)	36 (27)	133
Other ESS-codes (%)	232 (9)	880 (33)	987 (37)	1206 (19)	2660
**ICD-10 Diagnoses**					
Lower respiratory tract infections^c^ (%)	237 (24)	414 (42)	268 (27)	74 (8)	993
Upper respiratory tract infections^d^ (%)	89 (9)	344 (36)	368 (39)	148 (16)	949
Abdominal pain (R10) (%)	5 (2)	80 (24)	181 (54)	70 (21)	336
Acute appendicitis (%) (K35) (%)	3 (3)	35 (29)	73 (61)	9 (8)	120
Concussion (S06) (%)	5 (2)	39 (16)	122 (50)	80 (33)	246
Infectious gastroenteritis^e^ (%)	10 (2)	160 (36)	152 (34)	122 (28)	444
Other ICD-10 diagnoses (%)	235 (8)	1057 (35)	1125 (37)	636 (21)	3053
**Procedure codes**					
JEA: Appendectomy (%)	3 (3)	34 (29)	70 (60)	9 (8)	116
GXAV: Assisted ventilation (%)	21 (55)	13 (34)	4 (11)	0 (0)	38
**Hospitalized**^**f**^					
Entire population (%)	405 (13)	1254 (41)	1046 (34)	382 (12)	3087
Pediatric Medicine (%)	369 (17)	881 (41)	660 (30)	261 (12)	2171
Surgery^g^ (%)	25 (4)	274 (38)	328 (46)	90 (13)	717
**Long hospital stay**^**h,i**^					
Entire population (%)	244 (17)	647 (45)	424 (29)	138 (10)	1453
Pediatric Medicine (%)	231 (21)	470 (43)	287 (26)	107 (10)	1095
Surgery^g^ (%)	7 (3)	113 (48)	101 (43)	16 (7)	237
**Referral to PICU**^**j,k**^					
Entire population (%)	45 (43)	43 (41)	15 (14)	3 (3)	106
Pediatric Medicine (%)	43 (47)	35 (38)	13 (14)	1 (1)	92
Surgery^f^ (%)	2 (15)	7 (54)	2 (15)	2 (15)	13

^a^ *ESS* Emergency sign and symptoms, ^b^*ICD-10* International classification of diseases, 10th revision, ^c^ICD-10-codes: J09-J18, J20-J22 and J40-J47; ^d^ICD-10-codes: J00-J06 and J30-J39, ^e^ ICD-10-codes A00-A09, *p < 0.001 compared with Pediatric Medicine, ^f^Missing = 19, ^g^Surgery = general and neurosurgery, ^h^defined as hospital time (hours) > mean, ^i^Missing = 381 ^j^*PICU* Pediatric intensive care unit, ^k^Missing = 381

In all, 49% of the triaged children were hospitalized at the Pediatric Department at St. Olavs hospital, and 51% were treated as outpatients  (Table 1). More boys (53%) than girls (47%) (*p* = 0.006) were hospitalized. The excluded children had less surgical complaints compared to the included children (*p* < 0.001). In the entire population and all sub disciplines, a higher priority rate was significantly associated with hospitalization to wards, a long hospital stays and referral to the PICU compared to the reference category with low priority (green category) (Table 2). This association increased in strength according to higher priority levels (p-trend < 0.001). Compared to those with lowest priority, those with the highest priority were approximately five times more likely to be admitted, four times more likely to have a long hospital stay, and over 30 times more likely to be admitted to the PICU (Table 2). In the entire population, 54% of the hospitalized children, 61% of the children with long hospital stay and 83% with PICU referral were triaged to high priority in the PED (red or orange) (sensitivity). In all, 66% of the children who were not hospitalized, 62% with a short hospital stay and 57% without PICU referral were triaged to low priority ratings (yellow or green) (specificity). For the same three proxy variables, 34, 38 and 43%, respectively, of the children who were triaged to a high priority rating were overtriaged, and 46, 39 and 17%, respectively, of the children triaged to low priority ratings were undertriaged.

**Table 2:** Associations between triage priority levels and major outcomes among pediatric emergency patients expressed as odds ratio (ORs) with 95% confidence intervals (CIs^*^)

	All patients (***n*** = 6368)		Medicine (***n*** = 4727)		Surgery^**a**^ (***n*** = 1209)	
	Hospitalized^b^ (*n* = 3087)		Hospitalized^b^ (*n* = 2171)		Hospitalized^b^ (*n* = 717)	
	N (%)	OR (95% CI)	N (%)	OR (95% CI)	N (%)	OR (95% CI)
Green	382 (12)	ref.	261 (12)	ref.	90 (13)	ref.
Yellow	1046 (34)	1.7 (1.5–2.0)	660 (30)	1.5 (1.3–1.8)	328 (46)	2.3 (1.7–3.1)
Orange	1254 (41)	3.0 (2.6–3.5)	881 (41)	2.8 (2.4–3.4)	274 (38)	3.1 (2.3–4.3)
Red	405 (13)	5.0 (4.1–6.2)	369 (17)	5.1(4.1–6.5)	25 (3.5)	6.2 (2.7–14.3)
P-trend		< 0.001		< 0.001		< 0.001
	Long hospital stay^c,d^ (*n* = 1453)		Long hospital stay^c,d^ (*n* = 1095)		Long hospital stay^c,d^ (*n* = 237)	
Green	138 (10)	ref.	107 (10)	ref.	16 (7)	ref.
Yellow	424 (29)	1.5 (1.3–1.8)	287 (26)	1.4 (1.2–1.6)	101 (43)	2.0 (1.4–2.7)
Orange	647 (45)	2.6 (2.2–3.0)	470 (43)	2.5 (2.1–3.0)	113 (48)	2.4 (1.7–3.3)
Red	561 (17)	4.2 (3.4–5.3)	231 (21)	4.5 (3.6–5.8)	7 (3)	7.5 (2.5–22.1)
P-trend		< 0.001		< 0.001		< 0.001
	Referral to PICU^e,f^ (*n* = 106)		Referral to PICU^e,f^ (*n* = 92)		Referral to PICU^e,f^ (*n* = 13)	
Green	3 (3)	ref.	1 (1)	ref.	2 (15)	ref.
Yellow	15 (14)	2.4 (0.7–8.3)	13 (14)	6.5 (0.8–50.0)	2 (15)	0.5 (0.1–3.4)
Orange	43 (41)	8.1 (2.5–26.3)	35 (38)	19.7 (2.7–144.0)	7 (54)	2.2 (0.5–10.4)
Red	45 (43)	33.8 (10.5–109.2)	43 (47)	75.3 (10.3–548.5)	2 (15)	8.0 (1.1–58.7)
P-trend		< 0.001		< 0.001		0.02

^a^Surgery = general surgery and neurosurgery, ^b^Missing = 19, ^c^defined as hospital time (hours) > mean, ^d^Missing = 381, ^e^*PICU* Pediatric intensive care unit, ^f^Missing = 19, ^*^data is adjusted for age (continuous variable)

### Pediatric medical patients

Among pediatric medical patients 11% had a red priority, 34% had an orange priority, 36% had a yellow priority and 19% had a green priority (Table 1). Among pediatric patients 46% were hospitalized, and among pediatric patients with red triage 69% were hospitalized (Table 1). Further 23% had a long hospital stay and 2% were referred to PICU (Table 1). Most patients got ICD-10 diagnoses and ESS codes related to respiratory complaints (Table 1). As demonstrated in Table 2 there was increasing risk of hospitalization, having a long hospital stay and referral to the PICU according to priority level (p trend all; < 0.001). Compared to patients with lowest priority, those with highest priority were four to five times more likely to be hospitalized and have a long hospital stay (Table 2). They were further approximately 75 times more likely to be admitted to the PICU, compared to patients with lowest priority (Table 2). Patients with lower respiratory tract infection were more likely to have a high priority, compared to patients with upper respiratory tract infection (Table 3). Patients with gastroenteritis were more likely to have a high priority compared to patients with constipation (Table 3).

**Table 3:** Comparisons of triage priority levels in selected ICD-10^a^ diagnoses of various medical severity, expressed as odds ratio with 95% confidence interval and adjusted by age^*^

	ICD-10^a^ diagnoses	
	Lower vs. upper respiratory tract infection	
Green (ref.)		
Yellow	1.5	(1.1–2.0)
Orange	2.4	(1.8–3.3)
Red	5.4	(3.7–7.9)
P-trend		< 0.001
	Gastroenteritis vs. constipation	
Green (ref.)		
Yellow	1.1	(0.7–1.8)
Orange	2.1	(1.3–3.4)
Red	4.9	(0.9–37.5)
P-trend		< 0.001
	Appendicitis vs. constipation	
Green (ref.)		
Yellow	4.3	(1.9–9.7)
Orange	5.0	(2.0–12.3)
Red	7.0	(1.0–50.4)
P-trend		< 0.001
	Appendicitis vs. abdominal pain	
Green (ref.)		
Yellow	2.7	(1.3–5.9)
Orange	3.6	(1.6–8.2)
Red	6.5	(1.2–35.8)
P-trend		< 0.001

^a^*ICD-10* International classification of disease, 10th edition, ^*^data is adjusted for age (continuous variable)

### Pediatric surgical patients

Among general surgical and neurosurgical patients, 3% had a red triage, 33% had an orange triage, 44% had a yellow triage and 20% had a green triage (Table 1). Among the general surgical and neurosurgical patients 55% were hospitalized, and among patients with red priority 74% were hospitalized (Table 1). Further 18% had a long hospital stay and 0.1% were referred to PICU (Table 1). There was a significant association between priority level and hospitalization, having a long hospital stay and referral to the PICU (Table 2). These associations increased in strength according to priority level (Table 2; p-trend all < 0.001). Compared to patients with the lowest triage, patients with the highest triage had six times increased risk of hospitalization, seven times increased risk of having a long hospital stay and eight times more risk of being admitted to the PICU (Table 2). Patients with acute appendicitis were more likely to have a high priority compared to patients with constipation and abdominal pain (Table 3).

## Discussion

In the present study we found that a high RETTS-P priority rate was significantly associated with hospitalization, a long hospital stay and referral to the PICU compared to patients with low priority. This association increased in strength according to higher priority level. The data indicated that more severe conditions were higher prioritized than less severe conditions for both medical and surgical patients. This is the first study to investigate the validity of RETTS-p. Two previous studies have shown that RETTS is a valid tool among adults in Sweden [7] and Denmark [12].

Approximately one of ten children had a life-threatening condition and were triaged red in need of urgent intervention, one third were triaged orange implying examination within 20 min, one third were yellow in need of evaluation within 2 h, and one fifth received the lowest urgency level green with until 4 h waiting time. Interestingly, triage ratings varied between disciplines. Medical patients were more likely to have a high triage priority compared to the other specialties. This could suggest that RETTS-p have systematic errors in the way it prioritizes children. However, our further analyzes suggest that this is not the case. As medical patients also were more likely to have a long hospital stay and to be referred to the PICU, it is likely that they more often were severely ill than the surgical patients, and hence that the triage was correct. Furthermore, for both medical and surgical patients, those with more severe diagnosis at discharge and those receiving advanced treatment had a higher priority when triaged at the PED. For example, those with a lower respiratory tract infection were higher prioritized than children with upper respiratory tract infection. Also, children with appendicitis were higher prioritized than children with constipation and abdominal pain. These factors indicate that in the PED RETTS-p prioritized the children correctly.

We found that approximately one third of the referred children was overtriaged; e.g. 31% of the pediatric patients with red priority were not hospitalized. This is probably explained by the fact that these patients predominantly represented children with airway complaints, which needs promptly medical attention although the distress often is reversible with treatment. In contrast to the medical patients, surgical patients were likely to be admitted to hospital regardless of priority, resulting in relatively high rates of undertriage. We interpret that this finding reflects the need to monitor surgical patients in case they need surgery or develop complications at a later stage. As such, it seems that there are different traditions and procedures in treating medical and surgical patients, but that RETTS-p prioritize patients appropriate in the PED according to their medical state. Considering these inconsistencies, the use of hospitalization and length of hospital stay for validation may be questionable, but the association between high priority and these proxy variables on a group level is undisputable.

Strengths of the present study are the population-based design with children referred from primary health care, and large cohort size with a broad specter of pediatric medical and surgical diagnoses. Weaknesses are a retrospective design, and the fact that the proxy variables we have used could be influenced by other factors than illness severity. E.g. we only found moderate sensitivities and specificities for hospitalization and length of hospital stay, proxy variables that may be influenced by living conditions, care takers and travel distance to hospital etc. [13]. A more robust proxy variable might be referral to the PICU, as it is in general less affected by these factors, and reflects severe illness with need of intensive care, continuous observation and high resource use [13]. Indeed, we found that RETTS-p identified need of PICU referral with a higher sensitivity of 83%. We do not have any information regarding whether RETTS-p prioritized children with chronic illnesses differently from otherwise healthy children. Seiger et al. [11] demonstrated that children with chronic illnesses were in greater risk of being undertriaged by the Manchester triage system (MTS). We would like to think that RETTS-p might be a more sensitive triage tool in this aspect, as it includes ESS scores which gives a higher triage if the child has certain chronic condition. However, we have not compared RETTS-p with a triage tool that does not include ESS scores. At last, we did not adjust for possible confounding factors other than the children’s age. We do not have data regarding chronic illness, travel distance to hospital and socioeconomic status of the parents, which might have influenced the results.

Comparing various triage priority systems is a challenge due to different populations and health care systems, and the use of various proxy variables for true medical urgency [14]. In Norway, children are referred to PEDs after evaluation at general practitioners and physicians at municipal EDs. Recently Engan and al. studied a Norwegian modified version of the South African triage scale (SATS), but this study only included children with pediatric medical complaints [15]. In this study more patients received a green triage score and fewer patients received an orange triage score than the present study [15]. Using hospitalization as the sole proxy variable for true medical urgency, they report a higher sensitivity of 74% but a lower specificity of 48%, compared with our current findings on RETTS-p [15].

## Conclusion

The results of the present study indicate that RETTS-p has a high validity. We and others have previously shown that RETTS is a reliable triage system. Further studies should confirm our findings. Especially there is a need for prospective studies and studies that evaluate whether RETTS-p prioritize children with chronic illnesses different from otherwise healthy children.

## Data Availability

The dataset generated and/or analyzed during the current study is available from the corresponding author on reasonable request.

## Abbreviations

ED: Emergency department
ESS: Emergency signs and symptoms
GCS: Glasgow coma scale
ICD-10: International classification of disease, 10th edition
MTS: Manchester triage system
PED: Pediatric emergency department
PICU: Pediatric intensive care unit
RETTS-p: The rapid emergency triage and treatment system for children
VP: Vital parameters
